# Decomposing the Gap in Childhood Undernutrition between Poor and Non–Poor in Urban India, 2005–06

**DOI:** 10.1371/journal.pone.0064972

**Published:** 2013-05-29

**Authors:** Abhishek Kumar, Aditya Singh

**Affiliations:** 1 International Institute for Population Sciences, Deonar, Mumbai, India; 2 Global Health and Social Care Unit, School of Health Sciences and Social Work, University of Portsmouth, Portsmouth, United Kingdom; Indiana University, United States of America

## Abstract

**Background:**

Despite the growing evidence from other developing countries, intra-urban inequality in childhood undernutrition is poorly researched in India. Additionally, the factors contributing to the poor/non-poor gap in childhood undernutrition have not been explored. This study aims to quantify the contribution of factors that explain the poor/non-poor gap in underweight, stunting, and wasting among children aged less than five years in urban India.

**Methods:**

We used cross-sectional data from the third round of the National Family Health Survey conducted during 2005–06. Descriptive statistics were used to understand the gap in childhood undernutrition between the urban poor and non-poor, and across the selected covariates. Blinder–Oaxaca decomposition technique was used to explain the factors contributing to the average gap in undernutrition between poor and non-poor children in urban India.

**Result:**

Considerable proportions of urban children were found to be underweight (33%), stunted (40%), and wasted (17%) in 2005–06. The undernutrition gap between the poor and non-poor was stark in urban India. For all the three indicators, the main contributing factors were underutilization of health care services, poor body mass index of the mothers, and lower level of parental education among those living in poverty.

**Conclusions:**

The findings indicate that children belonging to poor households are undernourished due to limited use of health care services, poor health of mothers, and poor educational status of their parents. Based on the findings the study suggests that improving the public services such as basic health care and the education level of the mothers among urban poor can ameliorate the negative impact of poverty on childhood undernutrition.

## Introduction

According to United Nations (2012) estimates, the world urban population has surpassed rural population. About 52% (3.6 billion) of the world's population live in urban areas. By 2030 nearly 5 billion people will be living in urban areas [1]. Much of this urbanization is predicted to take place in the developing world, with Asia and Africa being the major contributors [2]. Like many other developing countries, India has also experienced rapid urbanization in last few decades. Recent estimates show that about 31% of the Indian population were living in urban areas in 2011, which is almost five times higher than in 1951 [3]. Though the level of urbanization in India is low, the country has the second largest urban population in the world with 377 million people living in urban areas and it is expected that 586 million population of the country will be residing in urban areas by 2030 [1].

The explosive growth of urban population in the developing countries is mainly fuelled by poverty led rural to urban migration. Accompanying this phenomenon is increasing urban poverty. For instance, according to the Urban Poverty in India report (2009), 26% of total urban population of India lives below the poverty line [4]. Moreover, the ratio of urban poverty in some of the populous states is higher than that of rural poverty [5]. Majority of the urban poor reside in overcrowded slums, which are characterised by extremely unhealthy living conditions — uncollected garbage, unsafe water, poor drainage and open sewers, poor health services and public infrastructure, which in turn, worsen their susceptibility to various health problems. For instance, about 57% of the urban population in Mumbai, India, live in slums [6], [7].

Increasing levels of urban poverty challenge the commonly held assumption that the economic condition of urban populations are superior to those of rural dwellers. Rapid growth in urban population has not been matched by an adequate expansion of sanitation, health services, and livelihood opportunities [8], [9]. Consequently, the urban advantage in health has faded in recent decades. Several studies in recent years have documented the high risk of maternal and child mortality among the urban poor in developing countries [10], [11], [12], [13], [14]. Childhood undernutrition is no exception to this. Newly assembled evidence from developing countries indicates that the locus of poverty and malnourishment is gradually shifting from rural to urban areas, as the number of urban poor and undernourished are increasing more rapidly than the rural ones [15]. Though the average childhood nutritional status is better in urban areas, the economic inequality in childhood nutritional status is higher in urban than rural areas of developing countries [16]. Using the Demographic and Health Survey (DHS) datasets from ten developing countries, a study has shown that the socioeconomic gradient in childhood stunting is indeed higher in urban areas than in rural areas [17]. Another study based on Sub-Saharan African countries has found that socioeconomic inequalities in childhood undernutrition are to a large extent, higher in cities than in rural areas [18]. The worst childhood nutritional status among the urban poor is attributed to their dismal living conditions, income constraints, and price barriers which limit the advantage that poor can reap from better food supply in urban areas [19]. In addition, the deficient nutritional status of urban poor children is caused by the repeated episodes of parasites or other childhood diseases like diarrhoea, which results from poor sanitation and unhygienic condition [20].

As noted earlier, the evidence show the enormous economic disparity in childhood nutritional status within urban areas of the developing countries, but the issue is poorly researched in India. Recent studies have examined the inequality in childhood undernutrition and found that the economic status of households is the prime contributor in explaining the inequality in childhood undernutrition in urban India [21], [22]. A few studies have also highlighted the growing poor/non-poor gap in the utilization of maternal healthcare services and child health in urban India [23], [24]. However, none of these studies explains the factors contributing to the gap in the utilization of the health services or child health between urban poor and non-poor. The present paper goes one step beyond to explain the factors contributing to the average distance between the urban poor and non-poor undernourished children in India. We focused on childhood undernutrition since it is a major public health challenge in the developing world and the Millennium Development Goal–1 (MDG1) exclusively focuses on reducing the proportion of underweight among children under age five years.

The present study aims to explain the factors contributing to the poor/non-poor gap in weight-for-age, height-for-age, and weight-for-height in urban India using cross-sectional data of the National Family Health Survey conducted during 2005–06. For the purpose, we used the Blinder-Oaxaca decomposition analysis which is useful in explaining the gap in outcome between the two population groups [25], [26]. An advantage of the decomposition analysis over the regression analyses is that it quantifies the contribution of factors that explain the average gap in an outcome between two groups. Additionally, the decomposition technique not only allows quantifying how much the difference in the distribution of health determinants contributes to the gap, but also investigates that how differences in the effects of the determinants, e.g. resulting from discrimination or inefficient use of the resources, contribute to the gap between poor and non-poor undernourished children in urban India. The evidence of the relative contribution of predictors that explain the gap in childhood undernutrition between the urban poor and non-poor may support policy makers in their efforts to reduce the poor/non-poor gap. To our knowledge there exists no published study that has accounted for the contribution of the factors explaining the poor/non-poor gap in childhood undernutrition in urban India by using by this type of decomposition technique.

## Methods

### Ethics Statement

The third round of the National Family Health Survey (NFHS–3) was conducted under the supervision of the International Institute for Population Sciences (IIPS), Mumbai, India – a regional centre of teaching, training, and research in population studies. The ORC Macro institutional review board approved the data collection procedures. A formal written consent was obtained and ethical issues were taken care of before interviewing the respondent in the survey. Moreover, this study is based on anonymous public use datasets with no identifiable information on the survey participants. Survey data are available upon the request on the official website of the institute at <www.measuredhs.com/data/dataset/India_Standard-DHS_2006.cfm?flag=0>.

### Data

This study used data from the third round of the National Family Health Survey conducted during 2005–06. The NFHS–3 is a large scale survey conducted on representative samples of households spanning the states and union territories of India. The survey provides reliable estimates of fertility, infant and childhood mortality, family planning, utilization of maternal and child health care services, childhood nutritional status at the country and state level and by urban-rural residence. The survey adopted multi-stage sampling design – a two-stage sampling design in rural areas and three-stage in urban areas. The NFHS–3 collected data using different interview schedules – household schedule, women/individual schedule, and men's schedule – from the sampled households. The household response rate was 98%, and the individual response rate was 95%. Details of sampling design, data collection tools, and sample size are given in the report of the NFHS–3 [27].

### Outcome Variables

The outcome variables in the present study are weight-for-age (underweight), height-for-age (stunting), and weight-for-height (wasting). We used the new reference population of the World Health Organization (WHO) standard to estimate all the three indicators for children below five years of age [28]. The NFHS–3 recorded information on all the three indicators for a total of 56,438 children below five years of age, 19,483 out of these belonged to urban areas. From this number, the analytical sample size of the present study was restricted to 15,241 children below five years of age after excluding missing and flagged cases, which comprise around 22% of the total urban sample (children less than five years) of the country.

### Explanatory Variables

The economic status of the household is the main exposure variable in the study. Like other DHS, NFHS–3 of India does not provide direct data on income or consumption. However, it provides information on a set of economic proxies such as housing quality, household amenities, consumer durables, and size of land holdings. Previous studies have used this information to assess the economic status of the households and to capture the economic differentials in the population and health outcomes by creating a composite measure called wealth index [29], [30], [31], [32], [33]. Following the standard and widely used approach, we used the Principal Component Analysis (PCA) to compute the wealth index for urban India based on a set of selected household economic proxies. Wealth index was computed separately for urban India considering the economic diversity between urban and rural areas [34]. The wealth index was subsequently divided into five quintiles – poorest, poorer, middle, richer, and richest – and the bottom two quintiles (lower 40%) were considered as poor and remaining three were as non-poor. This classification is consistent with previous studies [35].

The present study includes a number of socio-demographic and health care predictors in the analysis to explain the gap in childhood undernutrition between urban poor and non-poor. These variables are: sex of the child (male; female), age of the child (<12 months; 12–23 months; 24–35 months; ≥36 months), birth order of the child (1; 2; ≥3), size of the child at birth (large; average; small), mother's age at birth (<20 years; 20–24 years; 25–29 years; ≥30 years), mother's education level (uneducated; primary; secondary; >secondary), father's education level (uneducated; primary; secondary; >secondary), mother's exposure to media (unexposed; exposed), current working status of mother (not working; working), mother's body mass index–BMI (thin; normal; overweight/obese), mother's anaemic condition (anaemic; not anaemic), caste (SC/ST – Scheduled Caste and Scheduled Tribe; OBC – Other Backward Caste; Others – other castes), religion (Hindu; Muslims; others), childhood immunization (no immunization; full immunization), mother's visits for antenatal checkups (no; yes), institutional delivery (no; yes). We also included region (north; east; central; northeast; west; south) to capture the variation in health care provision and level of development across the regions of the country. All of these selected predictors were found to be significantly associated with childhood undernutrition in India [21], [35], [36].

The size of the child at birth is defined based on the mother's report of the baby's size at birth. Parental (both mother's and father's) education levels are classified on the basis of the number of years of schooling. The following categories were formed: no education (no schooling), primary education (1–5 years), secondary education (6–12 years) and, >secondary (>12 years of education). Body Mass Index (BMI) of the mother is defined as weight in kilograms divided by height in meters squared (kg/m^2^). We used the WHO classification to categorize the mothers into different BMI groups. Thus, BMI less than 18.5 is defined as thin, 18.5–24.9 is defined as normal, and a BMI of 25.0 and above defined as overweight/obese. We grouped obese and overweight together because very few (less than 2%) urban women were found to be obese in the 2005–06 survey. Based on the haemoglobin content in the blood of the respondent women, anaemia was classified into three levels of severity – mild anaemia (10–11.9 grams/deciliter), moderate anaemia (7.0–9.9 grams/deciliter), and severe anaemia (less than 7.0 grams/deciliter). The categorization is based on the guidelines adopted from the Centre for Disease Control and Prevention, 1998 [37]. In this study, mothers who suffered from any type of anaemia (mild, moderate, or severe) were considered as anaemic while others are considered as non-anaemic. Full immunization is defined as children who received one dose of BCG, three doses of DPT vaccine, three doses of polio vaccine, and one dose of measles vaccine; the others were defined as otherwise. Antenatal checkups is defined as at least four checkups in the first trimester of pregnancy, in line with the gold standard definition recommended by the World Health Organization [39]. Those mothers who received at least four checkups in the first trimester of pregnancy were considered under ‘yes’ category and those who had less than four checkups or did not have any checkups were classified as ‘no’. The regions were formulated by grouping of the states and were based on the regional classification of the NFHS [27].

### Statistical Analysis

Bivariate analysis was carried out to understand the gap in proportion of underweight, stunting, and wasting between the poor and non-poor children in urban India. In the bivariate analysis, we used the WHO standard to compute the z-scores for weight-for-age, height-for-age, and weight-for-height. Following the WHO guidelines, a child with a z-score of less than −2 standard deviations on these variables was classified as underweight, stunted, and wasted respectively (a binary outcome indicating 1 = underweight; 0 = otherwise and so on).

Descriptive analysis was carried out to compare the average gap in z-scores of weight-for-age, height-for-age, and weight-for-height between urban poor and non-poor across the selected covariates. Multiple linear regression was applied to examine the determinants of all the three indicators among the urban poor and non-poor. In the linear regression analysis, we used the z-scores of the weight-for-age, height-for-age, and weight-for-height. There were two reasons for using z-scores rather than using a binary outcome. *First*, z-scores are amenable to linear regression analysis and convey information on the extent of undernutrition rather than merely indicate whether a child is underweight or stunted or wasted. *Second*, it is essential for the Blinder–Oaxaca decomposition technique. In regression and decomposition analysis, the negative of the z-scores are used as the dependent variable. This facilitates interpretation as it has a positive mean and is increasing in undernutrition [40], [41].

### Blinder–Oaxaca Decomposition Method

Blinder–Oaxaca decomposition technique was used to decompose the gap in average z-scores of weight-for-age, height-for-age, and weight-for-height between urban poor and non-poor [32], [35], [40], [41], [42], [43]. This method allowed us to distinguish between two different sources of the gap in the outcomes between the poor and non-poor, which are (i) due to differences in the distribution of the determinants between the groups (poor and non-poor), (ii) due to differences in the effects of these determinants between the groups (poor and non-poor). More formally, assuming that the outcome is explained by only one determinant *x*, for each group (poor and non–poor) the outcome (z-scores of the indicators) ‘*y*’ could be expressed in a regression model in the following relationship (the effect of *x* is allowed to vary between the poor and non-poor).
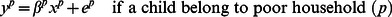
(1)

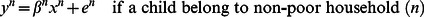
(2)The intercept term is also incorporated in the vector of *β* parameters and *e* is the error term. Then, the gap in the outcome between the two groups could be expressed as:
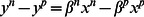
(3)Assuming the exogeneity, the conditional expectations of the error terms in equation (3) are zero [32]. Thus, the right hand side of the equation (3) can then be written as:
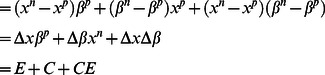
(4)Where *E* is “*endowment effect*” and expresses the contribution attributable due to the difference in distribution of the determinant *x* between urban poor and non-poor; *C* is *“coefficient effect”* and refers to the gap attributable to the difference of effect *x* between poor and non-poor; and *CE* is *“interaction effect”* and refers to the gap that is explained by the interaction between endowment and coefficient. There may be a chance of selection problem of children from poor households. In order to account for this, we used *lambda* option to check the difference in the group means and confirmed that the contribution of the predictors were generally similar to ones presented hereafter [32]. The analysis presented in the subsequent sections was carried out in STATA 10.0. The exposure variables were tested for possible multi-collinearity before putting them into the analysis. As the NFHS used multi-stage sampling design, standard errors were adjusted for weighting and clustering in all estimations. The detail of the sampling weight is given in the report of NFHS–3 [27].

## Results

Table 1 shows differences in some key indicators across children (aged 0–59 months) belonging to poor and non-poor households in urban India. In general, poor children belonged to less educated parents and were less likely to use health services. For instance, coverage of full immunization among poor children was about 43% while the corresponding figure among the non-poor children was 66%. Among the urban poor, 42% of mothers received at least four antenatal checkups and 47% delivered their births in health institutions while the corresponding figure among non-poor was 75% and 82% for antenatal checkups and institutional delivery respectively. In urban India, about two-fifths (38%) of poor mothers were undernourished (thin BMI) compared to little more than one-fifth (22%) of non-poor mothers.

**Table 1:** Differences in selected background characteristics among children across the poor and non-poor in urban India, 2005–06.

Variables	Poor	Non-poor	Total
Children of small size at birth	21.4	17.3	18.9
Mother educated with primary and above	48.4	87.0	71.6
Fathers educated with primary and above	66.8	94.6	83.6
Mothers age at birth <20 years	21.8	13.2	16.6
Mother exposed to media	75.5	96.3	88.1
Mother currently working	23.1	14.4	17.9
Mothers with thin BMI	37.8	21.8	28.2
Full immunization	42.6	66.3	57.4
Antenatal care checkups	41.6	75.4	63.1
Institutional delivery	46.6	81.5	67.6

### Result of the Descriptive Analysis

**Figure 1:**
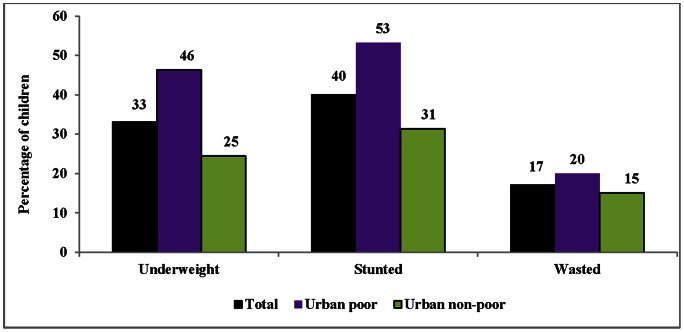
Percentage of undernourished children (aged 0–59 months) across the poor and non-poor in urban India, 2005–06.

Figure 1 shows differences in the prevalence (%) of underweight, stunting, and wasting among poor and non-poor children in urban India. The overall prevalence of underweight children in urban India was 33% in 2005–06. The corresponding prevalence of stunting and wasting was 40% and 17% respectively. The prevalence of all the three outcomes varied starkly between the urban poor and non-poor. More than two-fifths (46%) of poor children were underweight compared to only one-fourth (25%) among non-poor children. Similarly, about 53% of poor children were stunted compared to 31% among non-poor children. The prevalence of wasting was 20% and 15% among urban poor and non-poor respectively.

We also compared the mean of z-scores of weight-for-age, height-for-age, and weight-for-height between the urban poor and non-poor across the selected covariates (Table 2). In general, the mean (negative) of z-scores was lower among the urban poor than non-poor for all the three outcomes across the selected covariates. The mean z-scores of weight-for-age was lower (−1.80) among poor children with educated mothers than among non-poor children with educated mothers (−1.46). Similarly, among children with educated fathers, the mean was −1.85 among the urban poor compared to −1.48 among the non-poor. For children whose mothers had received four antenatal checkups, the mean of the z-scores was −1.80 among urban poor compared to −1.41 among non-poor children. A similar gap was observed for height-for-age and weight-for-height. We used the multiple regression analysis to find the determinants of weight-for-age, height-for-age, and weight-for-height across the urban poor and non-poor. Regression results are not discussed in detail for the sake of brevity but can be referred in **Appendix S1**.

**Table 2:** Mean differences in z-scores of weight-for age, height-for-age, and height-for-age among the children (0–59 months) between urban poor and non-poor across the selected indicators in India, 2005–06.

	Weight-for-age		Height-for-age		Weight-for-height	
Characteristics	Poor	Non-poor	Poor	Non-poor	Poor	Non-poor
Belonged to educated mother	−1.80	−1.46	−2.16	−1.68	−1.36	−1.29
Belonged to educated father	−1.85	−1.48	−2.24	−1.72	−1.38	−1.29
Mother exposed to media	−1.89	−1.49	−2.27	−1.72	−1.40	−1.30
Belonged to thin mother	−2.14	−1.48	−2.42	−1.90	−1.56	−1.48
Child is fully vaccinated	−1.79	−1.37	−2.44	−1.71	−1.35	−1.20
Antenatal care checkups	−1.80	−1.41	−2.09	−1.63	−1.47	−1.29
Institutional delivery	−1.84	−1.45	−2.18	−1.65	−1.43	−1.29
Total	−1.94	−1.49	−2.32	−1.74	−1.41	−1.30

### Result of the Decomposition Analysis

To quantify the contribution of selected predictors in explaining the poor/non-poor gap in the mean z-scores of weight-for-age, height-for-age, and weight-for-height, Blinder–Oaxaca decomposition analysis was used. To check for evidence of differences in the effects of the determinants, a regression analysis was carried out separately for weight-for-age, height-for-age, and weight-for-height z-scores on the determinants, interacting with the dummy variable separating the urban poor from the remaining urban population. The joint test on these interaction effect was significant (not shown), indicating that Blinder–Oaxaca decomposition is applicable in this context. Decomposition analysis was performed using STATA 10.0 routine ‘*decompose*’ program [32], [43].

The gap is decomposed into three parts; the *first* part is known as endowment effect and claimed the gap due to differences in the distribution of determinants between the urban poor and non-poor; the *second* part is the coefficient effect and claimed the gap due to the differences in the effect of determinants between the groups; and the *third* is interaction between the both – endowment effect and coefficient effect (interaction effect). However, the results are shown for endowment and coefficient effects only, as these two accounted for most of the gap in average z-scores of weight-for-age, height-for-age, and weight-for-height between the urban poor and non-poor. For instance, endowment effect accounted for 53% and coefficient effect accounted for 45% of the average gap in z-scores of weight-for-age between urban poor and non-poor while the contribution of the interaction effect was marginal and insignificant (Table 3). A similar pattern is observed in case of height-for-age and weight-for-height.

**Table 3:** Summary result of Oaxaca decomposition analysis showing the mean differences in z-scores of weight-for age, height-for-age, and height-for-age among poor and non-poor children in urban India, 2005–06.

	Weight-for-age		Height-for-age		Weight-for-height	
	Mean	P-value	Mean	P-value	Mean	P-value
Mean prediction high (Non-poor)	−1.54	0.00	−1.78	0.000	−1.32	0.00
Mean prediction low (Poor)	−2.03	0.00	−2.33	0.000	−1.54	0.00
Raw differential (Non-poor–poor)	0.48	0.00	0.54	0.000	0.22	0.00
Due to endowments	0.26	0.00	0.20	0.000	0.10	0.00
Due to coefficients	0.22	0.00	0.19	0.013	0.08	0.04
Due to interaction	0.01	0.80	0.16	0.082	0.04	0.12

Table 3 shows that children belonging to poor households had lower z-scores than those belonging to non-poor households in urban India, indicating that undernutrition was more prevalent among the urban poor than the non-poor. The average weight-for-age z-score was −2.03 among urban poor, compared to −1.54 among non-poor. Similarly, the average height-for-age z-scores was −2.33 among urban poor compared to −1.78 among non-poor, and weight-for-height z-score was −1.54 among poor and −1.32 among non-poor in urban India. The mean difference in z-scores between the groups was highly significant (p<0.001) for all three indicators.

Tables 4 and 5 show the contribution of endowment and coefficient effects respectively, in explaining the gap in average z-scores of weight-for-age, height-for-age, and weight-for-height between the poor and non-poor in urban India. A negative contribution indicates that the determinant was narrowing the gap between the poor and non-poor and vice-versa. Table 4 shows how differences in the distribution of each determinant contributed separately to the first part of the gap (endowment effect). In particular, the use of maternal health care services, poor health of the mother, and parental education (both mother and father) were the most important contributors explaining the gap in average z-scores of all the three indicators between the poor and non-poor. For instance, in the weight-for-age z-score, the contribution of four or more antenatal checkups was 22%, institutional delivery was 9%, mother's BMI was 19%, mother's education was 16%, and father's education was 13%. Similarly, in height-for-age z-score, four or more antenatal checkups contributed 20%, institutional delivery contributed 15%, full immunization contributed 16%, mother's BMI contributed 17%, mother's education contributed 11%, and father's education contributed 13% of the gap. For weight-for-height, the contribution of maternal education was highest (37%), followed by mother's BMI (34%), caste (28%), maternal and child health healthcare services and father's education. Factors like female child, higher birth order, mother's age at birth, exposure to the media, and religion appeared to minimize the gap in weight-for-height z-score between urban poor and non-poor.

**Table 4:** Contribution of differences in the distribution of the determinants of childhood undernutrition to the total gap between urban poor and non-poor in India, 2005–06.

	Weight-for-age		Height-for-age		Weight-for-height	
Variables	Z score gap	% Contribution	Z score gap	% Contribution	Z score gap	% Contribution
Child is female	NA	NA	NA	NA	−0.015	−14.7
Age of child	−0.015	−5.8	−0.036	−17.9	0.003	2.9
Birth order	0.010	3.9	0.018	9.0	−0.016	−15.7
Size of child at birth (average and above)	0.011	4.3	0.008	4.0	0.009	8.8
Mother's age at birth	−0.001	−0.4	0.008	4.0	−0.011	−10.8
Mother education (primary and above)	0.042	16.3	0.022	10.9	0.038	37.3
Father education (primary and above)	0.033	12.8	0.026	12.9	0.013	12.7
Exposure to media	−0.001	−0.4	0.012	6.0	−0.023	−22.5
Working status of mother	0.006	2.3	0.006	3.0	0.008	7.8
BMI of mother	0.049	19.1	0.035	17.4	0.035	34.3
Mother is anaemic	0.005	1.9	0.000	0.0	0.002	2.0
Religion	0.001	0.4	0.000	0.0	−0.020	−19.6
Caste	0.018	7.0	−0.002	−1.0	0.028	27.5
Full immunization	0.017	6.6	0.032	15.9	0.013	12.7
Antenatal care checkups	0.057	22.2	0.040	19.9	0.020	19.6
Institutional delivery	0.024	9.3	0.031	15.4	0.017	16.7
Regions	0.001	0.4	0.001	0.5	0.001	1.0
*Constant*	0.000	0.0	0.000	0.0	0.000	0.0
Total	0.257	100.0	0.201	100.0	0.102	100.0

NA: Not applicable in the analysis as not found significant in chi-squared test.

**Table 5:** Contribution of differences in the effect of the determinants of childhood undernutrition to the total gap between poor and non-poor in urban India, 2005–06.

	Weight-for-age		Height-for-age		Weight-for-height	
Variables	Z score gap	% Contribution	Z score gap	% Contribution	Z score gap	% Contribution
Child is female	NA	NA	NA	NA	−0.015	−18.5
Age of child	0.037	17.1	0.018	9.7	−0.030	−37.0
Birth order	−0.030	−13.9	0.027	14.5	−0.026	−32.1
Size of child at birth (average and above)	0.013	6.0	−0.029	−15.6	−0.001	−1.2
Mother's age at birth	0.074	34.3	−0.047	−25.3	0.026	32.1
Mother education (primary and above)	−0.031	−14.4	0.042	22.6	0.017	21.0
Father education (primary and above)	−0.035	−16.2	0.024	12.9	0.033	40.7
Exposure to media	0.060	27.8	0.032	17.2	0.027	33.3
Working status of mother	−0.028	−13.0	−0.040	−21.5	−0.019	−23.5
BMI of mothers	0.009	4.2	−0.009	−4.8	−0.019	−23.5
Mother is anaemic	0.073	33.8	0.005	2.7	0.029	35.8
Religion	0.003	1.4	0.011	5.9	0.006	7.4
Caste	−0.001	−0.5	0.021	11.3	−0.008	−9.9
Full immunization	−0.015	−6.9	−0.026	−14.0	0.023	28.4
Antenatal care checkups	−0.002	−0.9	0.024	12.9	−0.011	−13.6
Institutional delivery	0.012	5.6	0.047	25.3	0.032	39.5
Region	−0.041	−19.0	−0.013	−7.0	0.027	33.3
*Constant*	0.118	54.6	0.099	53.2	−0.010	−12.3
Total	0.216	100.0	0.186	100.0	0.081	100.0

NA: Not applicable in the analysis as not found significant in chi-squared test.

Table 5 shows the part of the gap that was accounted by different effect of the determinants (coefficient effect) between urban poor and non-poor. It shows that there were many offsetting factors such as – birth order, full immunization, current working status, and regions of the country. The negative contribution of mother's working status indicates that it has more protective effect on childhood nutrition among the urban poor. On the other hand, the size of a child at birth, mother's age at birth, mother's BMI, mother's exposure to media, and anaemic condition of the mother were among important contributors in explaining the gap in z-scores of weight-for-age, height-for-age, and weight-for-height between poor and non-poor children in urban India.

## Discussion

The findings show that a considerable proportion of urban children were undernourished in India during the period 2005–06. It is worth noting here that the prevalence of underweight and stunting among children in urban India is much higher than the average level of underweight in many of the South–East Asian and most African countries [44]. The prevalence of childhood undernutrition was higher among the children of urban poor than the non-poor. The pattern remained consistent across the selected background characteristics. The findings of this study are similar to that of the previous studies that revealed the enormous socioeconomic gap in health and health care utilization within urban settings [17], [24], [45], [46], [47], [48], [49]. Though the prevalence of childhood undernutrition was comparatively higher among the urban poor, the absolute higher prevalence among the urban non-poor children is also considerable. The higher prevalence of underweight and stunting among the non-poor children needs to be understood in the context of their food consumption pattern or environmental condition, which may not necessarily be a reflection of the asset-based economic status. However, the data do not permit assessment of the effect of these factors on childhood undernutrition among the urban non-poor.

The main purpose of this study was to disaggregate the effect of the determinants in explaining the gap in mean z-scores of weight-for-age, height-for-age, and weight-for-height between poor and non-poor children in urban India. We used Blinder–Oaxaca decomposition analysis to explain poor/non-poor gap in mean z-scores of all the three indicators. The use of this method allows quantifying the proportion of the gap attributable to the differences in the distribution of determinants, and also the part attributed to differences in the effect of determinants between the groups. The results reveal that the average gap in weight-for-age, height-for-age, and weight-for-height is attributed mainly due to differences in the distribution of determinants between the urban poor and non-poor.

The summary result of the decomposition analysis reveals that the average z-scores (negative) of weight-for-age, height-for-age, and weight-for-height are significantly lower among the poor children than the non-poor in urban India. The result of the decomposition analysis reflects more or less a similar pattern in the contribution of the factors explaining the gap in weight-for-age and height-for-age z-scores between urban poor and non-poor. However, we found a different picture in case of weight-for-height. A possible explanation could be because the weight-for-height is a measure of current nutritional status and likely to be affected by illness or infectious diseases just prior to the survey period.

The analysis revealed that the key factor that determined the nutritional status of poor urban children is the underutilization of maternal healthcare services. Inadequate utilization of antenatal care services, combined with limited information on good dietary and hygiene practices, may render many unidentified high–risk mothers prone to adverse pregnancy outcomes. These include low birth weight and poor nutritional status during infancy and childhood [50], [51]. Similarly, dropout from scheduled vaccination, which causes vitamin deficiency and lower immunity against the infectious diseases, may also lead to malnutrition. Previous studies from India suggest that measles play a significant role in the precipitation of malnutrition due to vitamin-A deficiency in preschool children [52]. In recent studies, the underutilization of MCH services among the urban poor is well documented [24], [49]. The lower use of health care services among the urban poor may be due to several barriers ranging from the cost of care, the cost of transportation, and low awareness of health-promoting behaviour. Moreover, health services in urban India suffer from skewed spatial distribution, shortage of health workers, poor infrastructure, overcrowding, and a weak referral system [53]. The lack of motivation among health providers and poor communication between healthcare providers and patients is also among important hurdles in utilization of MCH services by the urban poor in India [54], [55].

Lower BMI of poor mothers is another important factor which is responsible for the poor nutritional status of their children. The mother's poor health may affect her child's nutritional status right from the pregnancy stage to the early years of life due to low birth weight, limited breastfeeding, and improper care. There is a higher risk of intrauterine growth retardation in malnourished mothers, which results in low birth weight babies and undernutrition in infancy and childhood [56], [57], [58], [59]. In addition, the burden of poverty and hazardous living conditions may trap the malnourished mother in a vicious cycle of poor health for both herself and her child [60], [61].

The higher contribution of parental education (particularly the educational level of the father who is usually the breadwinner for the family) among the urban poor for all the three indicators can be understood through its lower income generation potential. Most of the poorly educated male parents are employed in the informal sector where the work is often irregular and the income is poor. Such economic status results in improper dietary practices, underutilization of health care services, and poor living conditions which are detrimental to child health [62]. The low levels of maternal education among the urban poor may limit her health-promoting behaviour through limited knowledge, poor nutritional practices, and limited autonomy within the household which has an important bearing on childhood nutritional status [63]. On the other hand, educated parents from non-poor households can reap the benefits of higher income by raising the nutritional status of their children through the purchase of food, medicines, and access to healthcare services.

The other contributors to endowment effect were higher birth order and caste. Higher birth order is an indication of the presence of siblings within the family and its positive contribution to weight-for-age and height-for-age may be understood through the ‘dilution effect’: as the number of children increases, family resources available to an individual child decrease [64]. A previous study from in India suggests that the children living with many siblings are more likely to be underweight [65]. The significant contribution of caste may be attributed to higher concentration of deprived caste groups (also known as SC/ST) in an unhealthy living environment as compared to the remaining population [41]. In contrast other caste groups are characterized by a relatively better socioeconomic status than the SC/ST and OBC population, and are thus at lower risk of childhood undernutrition [41].

The other part of the gap — caused by differences in the effect of determinants (coefficient), identifies the offsetting factors. Differences in the working status of the mother and full immunization reduce the average gap in weight-for-age, height-for-age, and weight-for-height among poor and non-poor children. This may be explained by the fact that these factors are associated with relatively greater household wealth and better use of health care services, both of which are favourable for better nutritional status of children living in poverty. On the other hand, differences in the effect of mothers' age at birth, exposure to media, and maternal health status disfavour the nutritional status of poor children.

## Conclusion

It is an accepted fact that rapid urbanization in India is characterized by a growing poor/non-poor divide in health and utilization of MCH services. The main findings from this analysis build a strong case for multipronged policies specifically targeted to improve the health of urban areas by reducing the gap between poor and non-poor. The result of the decomposition analysis clearly shows that the children belonging to poor households are undernourished not only because of poverty, but also due to limited use of maternal health care services and poor care resulting from lower educational status of parents and poor health of mothers. If policy makers want to reduce the gap in childhood undernutrition between the urban poor and the non-poor, the problem of low use of public services, such as antenatal checkups, full immunization coverage, and mother's education should be addressed among the urban poor. It may improve the nutritional status of poor children by ameliorating the negative impacts of poverty and could reduce the gap in childhood undernutrition between urban poor and non-poor in the country. Since, formal schooling offers a long-term solution, because there are long lags between the time when girls join primary school and the time they become mothers, an intermediate solution would be to focus on literacy training for older women [66]. Improving the ways health care systems effectively interact with illiterate mothers is another possible option that can be considered [41].

The findings of the study prioritize the need of the recently drafted National Urban Health Mission (NUHM). A major mandate of NUHM is to improve the health of the urban poor, particularly among slum dwellers and other disadvantaged sections, by enhancing equitable access to quality healthcare [9]. The NUHM should also address issues of mother's health, particularly of pregnant women to minimize the intergenerational transmission of undernutrition. These findings are not only applicable for reducing the poor/non-poor gap in childhood undernutrition, but also contribute to reducing the overall burden of childhood undernutrition (particularly of underweight and stunting) in urban India. In addition to targeting the urban poor, there is the need of a comprehensive nutritional strategy to reduce the burden of childhood undernutrition in urban India.

### Limitations of the study

A few limitations need to be considered while interpreting the result of the study. *First*, we have not considered twin children in the study. This could have affected the results slightly as twins are considered to be generally more vulnerable to poor nutrition and health, especially those born in poor households. *Second*, this study could not assess the contribution due to differences in access to (physical distance) and quality of the health care services availed by poor and non-poor population in urban India because variables capturing such characteristics were not available in the dataset used for the analysis. *Third*, this study examined the effects of only those variables which were measured in the survey, but the poor/non-poor gap in childhood undernutrition is also likely to be affected by the variation in unmeasured sources such as disease exposure, food intake etc, which were not captured. *Finally*, there may be state wise variations in factors affecting childhood undernutrition in urban areas which need further investigations.

## Supporting Information

Appendix S1 Multiple linear regression models showing determinants of childhood undernutrition between poor and non-poor in urban India, 2005–06.
(DOCX)
