# Supplementary material for: Decomposing the Gap in Childhood Undernutrition between Poor and Non–Poor in Urban India, 2005–06
Source: PLoS One. 2013 May 29;8(5):e64972. doi: 10.1371/journal.pone.0064972 (PMC3666977; doi:10.1371/journal.pone.0064972)
Supplement: Appendix S1 — Multiple linear regression models showing determinants of childhood undernutrition between poor and non-poor in urban India, 2005–06. (DOCX) [file pone.0064972.s001.docx]

**Appendix S1** Multiple linear regression models showing determinants of childhood undernutrition between poor and non-poor in urban India, 2005-06.

|  | Weight-for-age | | Height-for-age | | Weight-for-height | |
| --- | --- | --- | --- | --- | --- | --- |
|  | Poor | Non-poor | Poor | Non-poor | Poor | Non-poor |
| Sex of the child |  |  |  |  |  |  |
| Male® |  |  |  |  |  |  |
| Female | NA | NA | NA | NA | 0.073^c^ | 0.051^c^ |
| Age of the child |  |  |  |  |  |  |
| <12 months® |  |  |  |  |  |  |
| 12-23 months | 0.198^a^ | 0.152^a^ | 0.779^a^ | 0.455^a^ | –0.339^a^ | –0.264^a^ |
| 24-35 months | 0.299^a^ | 0.281^a^ | 0.887^a^ | 0.493^a^ | –0.432^b^ | –0.212^a^ |
| ≥36 months | 0.334^a^ | 0.289^a^ | 0.753^a^ | 0.338^a^ | –0.446^a^ | –0.186^a^ |
| Birth order |  |  |  |  |  |  |
| 1® |  |  |  |  |  |  |
| 2 | 0.065 | 0.088^b^ | 0.206^b^ | 0.172^a^ | –0.084 | 0.042 |
| ≥3 | 0.109^c^ | 0.090^b^ | 0.339^a^ | 0.153^b^ | –0.103^c^ | –0.005 |
| Size of child at birth |  |  |  |  |  |  |
| Large® |  |  |  |  |  |  |
| Average | 0.019 | 0.045 | –0.074 | 0.027 | 0.104^b^ | 0.048 |
| Small | 0.334^a^ | 0.345^a^ | 0.199^b^ | 0.246^a^ | 0.274^a^ | 0.209^a^ |
| Mother's age at birth |  |  |  |  |  |  |
| <20® |  |  |  |  |  |  |
| 20-24 | –0.029 | –0.010 | –0.244^a^ | 0.004 | 0.086 | 0.024 |
| 25-29 | –0.052 | –0.058 | –0.248^a^ | –0.061 | 0.134^c^ | –0.045 |
| ≥30 | –0.017 | –0.041 | –0.205^b^ | –0.055 | 0.272^b^ | 0.010 |
| Mother education |  |  |  |  |  |  |
| Uneducated® |  |  |  |  |  |  |
| Primary | –0.065 | –0.055 | –0.041 | –0.046 | –0.085 | 0.019 |
| Secondary | –0.070 | –0.100^b^ | –0.016 | –0.126^b^ | –0.073 | –0.027 |
| >Secondary | –0.006 | –0.257^a^ | –0.153 | –0.254^c^ | –0.093 | –0.087 |
| Father education |  |  |  |  |  |  |
| Uneducated® |  |  |  |  |  |  |
| Primary | –0.145^a^ | 0.043 | –0.089 | –0.085 | 0.015 | –0.135 |
| Secondary | –0.142^a^ | –0.012 | –0.077 | –0.119 | –0.047 | –0.054 |
| >Secondary | –0.189^b^ | –0.052 | –0.139 | –0.213 | –0.210^c^ | –0.036 |
| Mother’s exposure to media |  |  |  |  |  |  |
| Unexposed® |  |  |  |  |  |  |
| Exposed | –0.055 | –0.003 | –0.072 | –0.119 | 0.048 | 0.054 |
| Current working status of mother |  |  |  |  |  |  |
| Not working® |  |  |  |  |  |  |
| Working | –0.002 | 0.032 | 0.006 | 0.038 | 0.017 | 0.067 |
| BMI of mother |  |  |  |  |  |  |
| Thin® |  |  |  |  |  |  |
| Normal | –0.265^a^ | –0.250^a^ | –0.097^b^ | –0.083^b^ | –0.201^a^ | –0.184^a^ |
| Overweight/Obese | –0.390^a^ | –0.422^a^ | –0.117^b^ | –0.185^a^ | –0.348^a^ | –0.320^a^ |
| Anaemia among mother |  |  |  |  |  |  |
| No-anaemic® |  |  |  |  |  |  |
| Anaemic | 0.083^b^ | –0.013 | 0.024 | –0.001 | 0.049 | –0.046 |
| Religion |  |  |  |  |  |  |
| Hindu® |  |  |  |  |  |  |
| Muslim | 0.098^b^ | 0.004 | 0.124^b^ | 0.062 | 0.100 | –0.035 |
| Others | 0.103 | –0.082^b^ | 0.149 | –0.006 | 0.140 | 0.007 |
| Caste |  |  |  |  |  |  |
| SC/ST® |  |  |  |  |  |  |
| OBC | –0.054 | –0.059 | –0.108^c^ | –0.027 | 0.016 | –0.023 |
| Other | –0.154^a^ | –0.105^b^ | –0.173^b^ | –0.120^b^ | –0.113^b^ | –0.054 |
| Childhood immunization |  |  |  |  |  |  |
| No® |  |  |  |  |  |  |
| Full immunization | –0.075^c^ | –0.064^b^ | –0.129^b^ | –0.097^b^ | –0.037 | –0.080^b^ |
| Antenatal care checkups |  |  |  |  |  |  |
| No® |  |  |  |  |  |  |
| Yes | –0.101^a^ | –0.075^b^ | –0.142^b^ | –0.085^b^ | 0.009 | –0.022 |
| Institutional delivery |  |  |  |  |  |  |
| No® |  |  |  |  |  |  |
| Yes | –0.074^c^ | –0.040 | –0.095^c^ | –0.167^c^ | 0.063 | 0.076^c^ |
| Region |  |  |  |  |  |  |
| North® |  |  |  |  |  |  |
| Central | 0.223^b^ | 0.106^b^ | 0.082 | 0.125^b^ | 0.095 | 0.132^a^ |
| East | 0.112 | 0.003 | –0.067 | –0.061^b^ | 0.108 | 0.085 |
| Northeast | –0.119 | –0.171^a^ | –0.171^c^ | –0.103^c^ | –0.099 | –0.081 |
| West | 0.242^b^ | 0.087^b^ | 0.080 | 0.148^b^ | 0.135 | 0.001 |
| South | 0.134^c^ | –0.058 | 0.008 | –0.143^b^ | 0.083 | –0.003 |
| Constant | 1.944^a^ | 1.729^a^ | 2.012^a^ | 2.037^a^ | 1.678^a^ | 1.630^a^ |

Note: The models are adjusted for the states of the country.

®: Reference category. ^a^P<0.01; ^b^P<0.05; ^c^P<0.10.

NA: Not used in the analysis as not found significantly associated in chi-squared test.
